# A Temperature Compensation Method for Piezo-Resistive Pressure Sensor Utilizing Chaotic Ions Motion Algorithm Optimized Hybrid Kernel LSSVM

**DOI:** 10.3390/s16101707

**Published:** 2016-10-14

**Authors:** Ji Li, Guoqing Hu, Yonghong Zhou, Chong Zou, Wei Peng, Jahangir Alam SM

**Affiliations:** 1 Department of Mechanical and Electrical Engineering, School of Aerospace Engineering, Xiamen University, Xiamen 361005, China; 19920130154215@stu.xmu.edu.cn; 2 Fujian Wide Plus Precision Instruments Co. Ltd., Fuzhou 350015, China; zyh@wideplus.com (Y.Z.); c@wideplus.com (C.Z.); 3 Department of Mechatronics Engineering, School of Mechanical & Automotive Engineering, South China University of Technology, Guangzhou 510640, China; mepeng.wei@mail.scut.edu.cn (W.P.); mejahangir@scut.edu.cn (J.A.S.)

**Keywords:** piezoresistive pressure sensor, temperature compensation, hybrid kernel LSSVM, chaotic ions motion algorithm

## Abstract

A piezo-resistive pressure sensor is made of silicon, the nature of which is considerably influenced by ambient temperature. The effect of temperature should be eliminated during the working period in expectation of linear output. To deal with this issue, an approach consists of a hybrid kernel Least Squares Support Vector Machine (LSSVM) optimized by a chaotic ions motion algorithm presented. To achieve the learning and generalization for excellent performance, a hybrid kernel function, constructed by a local kernel as Radial Basis Function (RBF) kernel, and a global kernel as polynomial kernel is incorporated into the Least Squares Support Vector Machine. The chaotic ions motion algorithm is introduced to find the best hyper-parameters of the Least Squares Support Vector Machine. The temperature data from a calibration experiment is conducted to validate the proposed method. With attention on algorithm robustness and engineering applications, the compensation result shows the proposed scheme outperforms other compared methods on several performance measures as maximum absolute relative error, minimum absolute relative error mean and variance of the averaged value on fifty runs. Furthermore, the proposed temperature compensation approach lays a foundation for more extensive research.

## 1. Introduction

Due to some unsatisfied aspects in manufacturing processes, such as the inconsistent doping concentration, mismatched thermal expansion coefficient among packaging materials, and electronics performance being sensitive to the mutation of the ambient temperature, monocrystalline silicon piezo-resistive pressure sensors suffer from nonlinear input–output characteristics as ambient temperature changes [1].

In order to eliminate the crucial disturbance by temperature, a number of techniques were presented. Among all methodologies, there are two dominant ways: hardware compensation from measuring principle viewpoint and software compensation from algorithm viewpoint. The hardware compensation regularizes circuit structure to approach the ideal input–output characteristics [2,3,4]. The limitations of debugging difficulty in the actual manufacturing process, increased cost, and limited compensation precision restrict the utilization of hardware compensation. The temperature compensation, in a certain sense, is a function approximation problem. The software compensation, in the context of which is mainly composed of conventional mathematical computation and artificial intelligence. The conventional software compensation methods of look-up table, spline interpolation and surface fitting are based on function fitting and interpolation [5,6,7,8]. The methods derived from artificial intelligence include neural networks [9,10,11,12] and support vector machines [13,14,15]. Although the conventional methods have a lower degree of difficulty when implemented in sensor circuits, they may encounter some trouble, which would arise from data collection cost with the increasing requirement of compensation precision or ill-conditioning problems in solving normal equations. Generally, the neural networks are based on an empirical risk minimum (ERM) principle and deepest gradient descent iteration, which may result in some defects such as dimension curse, local minimum, under-fitting or over-fitting, requirement of an amount of training set data, etc. Vapnik [16] invented a brand new machine learning theory known as support vector machines (SVM), which control the model complexity by the Vapnik-Chevernenkis (VC) dimension. SVM mainly aims at resolving classification and function approximation problems with relatively small samples. The structural risk minimum (SRM) principle of SVM that considers both ERM and confidence intervals has more decent learning and generalization ability than neural networks. To simplify and accelerate the solving process of SVM, Suykens [17] introduced the least squares support vector machine (LSSVM), which converts the inequality constraints in traditional SVM to linear equations in the framework of the regularization theory. Although the LSSVM can perform regression with quite high precision, the kernel function and hyper-parameters of it decide the actual outcomes. Consequently, a convex combination of different valid kernels and a chaotic ions motion algorithm are put forward to identify a proper kernel function and to customize the best hyper-parameters for LSSVM. 

## 2. Temperature Effect on Piezoresistive Pressure Sensor

**Figure 1:**
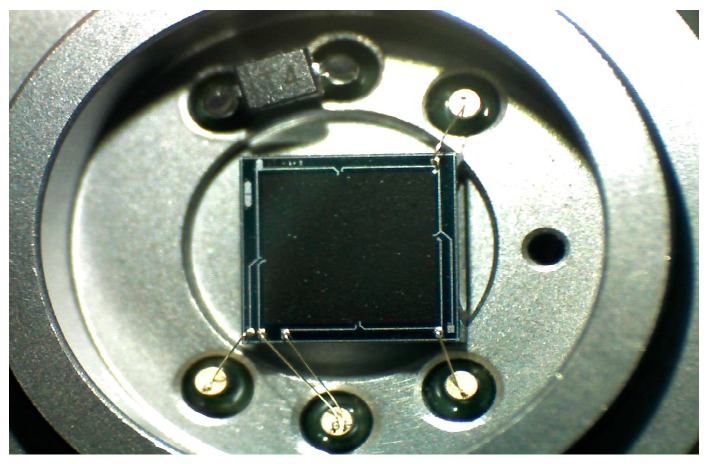
Packaging of the piezoresistive pressure sensor.

**Figure 2:**
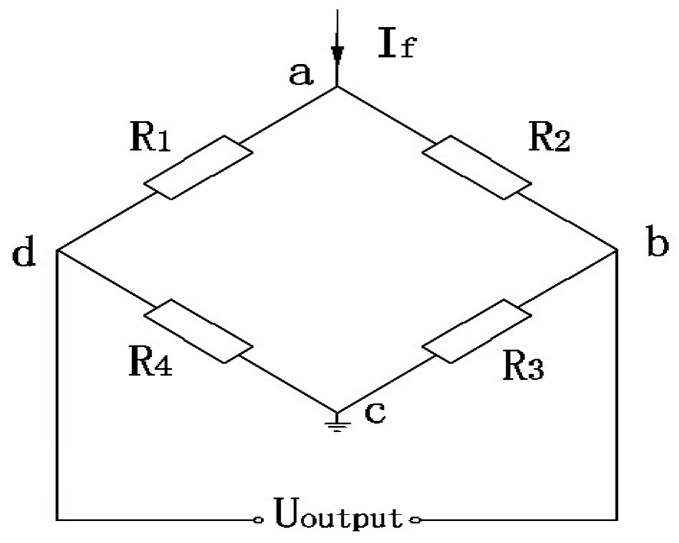
Configuration of the piezoresistive pressure sensor.

The operating principle of a piezoresistive pressure sensor is based on a piezoresistive effect that converts physical pressure signal to electrical signal. The packaging of the piezoresistive pressure sensor used in this article is shown in Figure 1. Generally, a symmetrical Wheatstone bridge consisting of four resistances (R_1_ = R_2_ = R_3_ = R_4_ = R) is the configuration of the piezoresistive pressure sensor, which is illustrated in Figure 2. 

By analyzing the Wheatstone bridge in Figure 2, the output voltage can be expressed as in the following [18]:

(1) $$U_{output}=I_{f}\times \Delta R=I_{f}R\pi \sigma ,$$

where $I_{f}$ is a constant current supplied by a steady current source, R is the value of resistance in each bridge arm, π is the piezoresistive coefficient of silicon, and σ is the stress applied on the sensor.

Ideally, $U_{output}$ is linearly proportional to σ if R and π are both constants. Nevertheless, the ambient temperature has a significant influence, namely the temperature effect, on R and π in reality. The bridge arm resistance and the piezoresistive coefficient are denoted as in the following [19]:

(2) $$R=R_{0}\left(1+\alpha \Delta T\right),$$

(3) $$\pi =\pi_{0}\left(1+\beta \Delta T\right),$$

where $R_{0}$ and $\pi_{0}$ are the resistance value and piezoresistive coefficient at room temperature, α and β are temperature coefficients of R and π, and ΔT is the ambient temperature variation. An additional stress will be generated between the support beam and substrate of the sensor when the ambient temperature varies. The additional stress quantity can be represented as [20]:

(4) $$\Delta \sigma =\frac{\left(\alpha_{s}-\alpha_{g}\right)E_{0}\Delta T}{1+\mu },$$

where $\alpha_{s}$ and $\alpha_{g}$ are the thermal expansion coefficients of silicon and glass, respectively, and $E_{0}$ is the temperature coefficient of silicon at Kelvin temperature. Substituting Equations (2)–(4) into Equation (1), it should take an offset item θ, which depicts the error among every bridge arm resistance in fabrication process, and then the complete expression can be calculated as in the following:

(5) $$U_{output}=I_{f}R_{0}\pi_{0}\left[1+\left(\alpha +\beta +\alpha \beta \Delta T\right)\Delta T\right]\left[\sigma +\frac{\alpha_{s}-\alpha_{g}E_{0}\Delta T}{1+\mu }+\theta \right].$$

According to the above analysis, the output voltage is not linearly scaled with the ambient temperature change. Additionally, some unavoidable reasons such as the asynchronously performance change of electronics with ambient temperature mutation and the different doping concentration in forming the bridge arm resistance, which directly determines α and β, also make the nonlinear relationship between $U_{output}$ and ΔT more complicated.

## 3. Hybrid Kernel LSSVM

### 3.1. LSSVM

Assuming a sample as (*x*_1_, *y*_1_), (*x_i_*, *y_i_*), (*x_m_*, *y_m_*) ∈ ***R**^n^* × ***R*** (*x_i_* is input, *y_i_* is output) and these data pairs are all generated from an independent and identical distribution. According to the Cover theorem, inputs from a nonlinear separable space become more separable by mapping them into a higher dimensional space. LSSVM implements the work with a kernel trick as well as SVM, and the discriminant function can be depicted as in the following [21]:

(6) $$F=\left\{f|f\left(x\right)=\omega^{T}\cdot \varphi \left(x\right)+b,\omega \in R^{n}\right\},$$

where ω is a weight vector in hyperplane space, *b* is the bias and φ(x) is the kernel function that satisfies the Mercer condition.

The regression problem in Equation (6) can be equally described as a convex optimization by adding a regularization item:

(7) $$\begin{array}{c} min\frac{1}{2}\omega^{T}\omega +\frac{1}{2}C\overset{n}{\underset{i=1}{\sum }}e_{i}^{2}\\ s.t.\;y_{i}=\omega^{T}\cdot \varphi \left(x\right)+b+e_{i},i=1,2,\cdots ,n. \end{array}$$

Then, constructing a Lagrange function:

(8) $$L\left(\omega ,b,e_{i},\alpha \right)=\frac{1}{2}\omega^{T}\omega +\frac{1}{2}C\sum_{i=1}^{n}e_{i}^{2}-\sum_{i=1}^{n}\alpha_{i}(\omega^{T}\cdot \varphi \left(x\right)+b+e_{i}-y_{i}).$$

According to the Karush-Kuhn-Tucker (KKT) conditions, taking the derivative with respect of all variables of the Lagrange function:

(9) $$\frac{\partial L}{\partial \omega }=0,\frac{\partial L}{\partial b}=0,\frac{\partial L}{\partial e_{i}}=0,\frac{\partial L}{\partial \alpha_{i}}=0.$$

The results are represented as follows:

(10) $$\begin{array}{c} \omega =\overset{n}{\underset{i=1}{\sum }}\alpha_{i}\varphi \left(x_{i}\right),-\overset{n}{\underset{i=1}{\sum }}\alpha_{i}=0,\\ \alpha_{i}=Ce_{i},\omega^{T}\cdot \varphi \left(x\right)+b+e_{i}-y_{i}=0. \end{array}$$

From the vector calculation perspective, Equation (10) can be translated in a form of a system of linear equations as:

(11) $$\left|\begin{array}{cc} 0 & I\\ I & \Omega +\gamma^{-1}I \end{array}\right|\cdot \left|\begin{array}{c} b\\ \alpha \end{array}\right|=\left|\begin{array}{c} 0\\ y \end{array}\right|,$$

where *I* is a column vector with each element of it equals to one, and Ω is an *n* × *n* matrix constructed by kernel mapping and data point:

(12) $$\Omega_{ij}=\varphi {\left(x_{i}\right)}^{T}\varphi \left(x_{j}\right)=K\left(x_{i},x_{j}\right),\left(i,j=1,2,\cdots ,n\right).$$

Because *A* = Ω + $\gamma^{-1}I$ is a symmetric positive definite matrix, the solution of Equation (12) can be denoted as:

(13) $$b=\frac{E^{T}A^{-1}y}{E^{T}A^{-1}E},\alpha =A^{-1}\left(y-bE\right).$$

Finally, the regression model of LSSVM is:

(14) $$f\left(x\right)=\overset{n}{\underset{i=1}{\sum }}\alpha_{i}K\left(x,x_{i}\right)+b.$$

### 3.2. Hybrid Kernel Function

Since the kernel function is one of the critical factors that contributes to approximation capability, a reasonable choice of kernel function is necessary. Actually, existing useful kernel functions that meet the Mercer kernel condition, namely admissible kernels, have various properties that differ from each other. Nevertheless, a majority of kernel functions belong to two types, namely local kernel function and global kernel function. Local kernel function is of desirable learning ability for it merely affects data points in the neighborhood of the test points. A typical local kernel function is the Radial Basis Function (RBF) kernel, which is defined as:

(15) $$K_{RBF}=exp\left(-\frac{{\left\|x-x_{i}\right\|}^{2}}{2\sigma^{2}}\right),$$

where the width σ is the only kernel parameter.

All test points in a global kernel function act as key points to determine the data points, and this property is devoted to excellent generalization ability in the context of LSSVM. The polynomial kernel function, a typical global kernel function, is defined as:

(16) $$K_{poly}={\left(x\cdot x_{i}+t\right)}^{p},$$

where *t* and *p* are the kernel parameters represent the bias and power of the polynomial, respectively.

Without any loss of generality, a convex combination of a radial basis function (RBF) kernel marked as K_RBF_ and a polynomial kernel marked as *K_poly_* is an admissible kernel holds the Mercer kernel condition as well [22], which is defined as:

(17) $$K_{h}=\lambda K_{poly}+\left(1-\lambda \right)K_{RBF},$$

where λ is a weight coefficient locates in the interval of (0, 1).

## 4. Chaotic Ions Motion Algorithm

### 4.1. Ions Motion Algorithm

Javidy [23] proposed a kind of population-based stochastic optimization methods called ions motion algorithm that achieves a balance between the confliction of diversification and intensification in the searching of optimal solution. Ions motion algorithm is inspired by the fact that ions with similar charges tend to repel, whereas ions with opposite charges attract each other. Each ion in the solution space stands for a candidate solution. The solution space is initialized randomly and divided into two groups: anions and cations. The repulsion/attraction force drives all ions moving across the solution space to find a better solution. To make the algorithm keep exploring and exploiting the solution space continuously, two stages including liquid phase (diversification) and crystal phase (intensification) are introduced.

The mathematical model of liquid phase is described as follows:

(18) $$AF_{i,j}=\frac{1}{1+e^{-0.1/AD_{i,j}}},$$

(19) $$CF_{i,j}=\frac{1}{1+e^{-0.1/CD_{i,j}}},$$

where $AD_{i,j}=\left|A_{i,j}-Cbest_{j}\right|$, $CD_{i,j}=\left|D_{i,j}-Abest_{j}\right|$, *i* indicates the ion index, *j* is the *j*th dimension of an individual ion, $AD_{i,j}$ is the distance between the *i*th anion and the best cation in *j*th dimension, $CD_{i,j}$ is the distance between the *i*th cation and the best anion in *j*th dimension, and $AF_{i,j}$ and $CF_{i,j}$ denote the attraction force of anions and cations separately. The force calculation is followed by a position updating as follows:

(20) $$A_{i,j}=A_{i,j}+AF_{i,j}\times \left(Cbest_{j}-A_{i,j}\right),$$

(21) $$C_{i,j}=C_{i,j}+CF_{i,j}\times \left(Abest_{j}-C_{i,j}\right),$$

where *i* and j also indicates the ion index and the dimension index, respectively.

According to Equations (18)–(21), ions in liquid phase always possess the exploring ability in the inherent diversity in the searching space. However, the diversity is gradually decreased along with iterations that would exactly lead the liquid phase to next phase, the crystal phase. The crystal phase is a certain way to jump out of local optima found by the liquid phase, which is depicted as follows (Algorithm 1):
**Algorithm 1***if (CbestFit* ≥ *CworstFit/2 and AbestFit* ≥ *AworstFit/2)*    *if rand() > 0.5*        *Ai* = *Ai* + *Φ1* * *(Cbest − 1)*    *else*        *Ai* = *Ai* + *Φ1* * *Cbest*    *end*    *if rand() > 0.5*        *Ci* = *Ci* + *Φ2* * *(Abest − 1)*    *else*        *Ci* = *Ci* + *Φ2* * *Abest*    *end*    *if rand() < 0.05*        *Re - initialized Ai and Ci*    *end**end*


The *CbestFit* and *CworstFit* are the fitness of the best and the worst cation, *AbestFit* and *AworstFit* indicates the fitness of the best and the worst anion, $\phi_{1}$ and $\phi_{2}$ are random numbers in the interval of [−1, 1], *rand* is a function that generates random numbers in [0, 1], and all random numbers used in the position updating in crystal phase obey the uniform distribution. 

### 4.2. Chaotic Initialization and Searching

Chaos is a nonlinear random-like deterministic bounded system, which is neither period nor converging [24]. Moreover, a chaotic system depends on its initial parameter and condition sensitively. The regular, random, ergodic and unpredictable essence of chaos makes it a reliable randomness source [25]. Instead of generating random sequence from the uniform distribution, the chaotic sequence provides a more effective searching plan for heuristic optimization algorithms [26]. The logistic map adopted in this article to generate solution sequence is described as follows:

(22) $$c_{i+1}=\mu \cdot c_{i}\left(1-c_{i}\right),i=1,2,\cdots ,m,$$

where μ is the control parameter, μ∈(2, 4], and *i* represents the iteration index. Suppose the initial value $c_{1}\in \left(0,\;1\right)$ and $c_{1}\notin \left\{0.25,\;0.5,\;0.75\right\}$, thus the chaotic behavioris ensured on the premise that μ=4.

The basic ideas of chaotic ions motion algorithm are described as follows:

(1) Chaotic initialization
  The initialization of the ions group is one of the key points regarding whether the convergence speed is acceptable and the final solution quality is pleased. Even though the initial position of each ions is random in original ions motion algorithm, ions might be located far away from the optimal ion. Chaotic initialization is for the purpose of increasing the diversity among the population and accelerating the convergence speed.
(2) Chaotic searching
  Restart with the last ion position in the latest generation and replace all other ion positions by generating a new chaotic sequence. Continuing to generate new ion positions by chaotic sequence rather than by probability distributions could avoid stagnation and speed the convergence in the optimization searching process.

### 4.3. Chaotic Ions Motion Algorithm Optimized Hybrid Kernel LSSVM (CIMA-Hybrid-LSSVM)

After assigning the kernel function of LSSVM, the remaining part that determines the temperature compensation’s performance is the hyper-parameters set of C, t, p, σ and λ. Parameter *C* determines the penalty of estimation errors. A large *C* not only assigns more penalties to errors but also allows higher generalization, while a small *C* has the opposite effect. Parameters *t* and *p* control the polynomial kernel function smoothness and fitness with given data. Similarly, parameter σ is responsible for the smoothness and fitness of RBF kernel function. Parameter λ weights the contribution of each kernel of the hybrid kernel function. All parameters are set in an empirical range: C∈(0.001, 10000), t∈(0.01, 200), p∈[1, 5], λ∈(0, 1).

The details of the proposed temperature compensation are as follows:

(1) Chaotic initialization
  - Chaotically distribute the seeds according to the dimension number of an ion by logistic map within the range of (0, 1).
  - Calculate the chaotic sequence with chaotic seeds until the chaotic sequence length reaches the ions population size.
  - Transform the chaotic sequence members into every parameter’s range, and take the transforming of C as an example:
    (23) $$C_{i}=C_{min}+Chaos_{i}\cdot \left(C_{max}-C_{min}\right),$$
    where *i* is the index of the chaotic sequence, $C_{min}$ is the lower limitation of *C*, and $C_{max}$ is the upper limitation of C.
(2) Generate the initial ion population by Equation (23) and divide it randomly into an anion group and a cation group at the same size.
(3) Evaluate each ion in liquid phase and rank them in terms of fitness. Record the best fitness and position of anion group, cation group and the ion population. If the best fitness of the ion population can neither satisfy the predetermined estimation precision nor reach the maximum searching generation number, then go to Step 4.
(4) Take the last seed of the chaotic sequence generated by Step 1 and repeat the procedure as in Step 1 to obtain a new chaotic sequence. Compute each ion fitness according to the rules stated in crystal phase with the new chaotic sequence. The computation comes to an end as the best fitness satisfies the predetermined estimation precision or reaches the maximum searching generation number. If the stop criterion can not be met, then take the last ion position as the chaotic seed and go back to Step 1.

**Figure 3:**
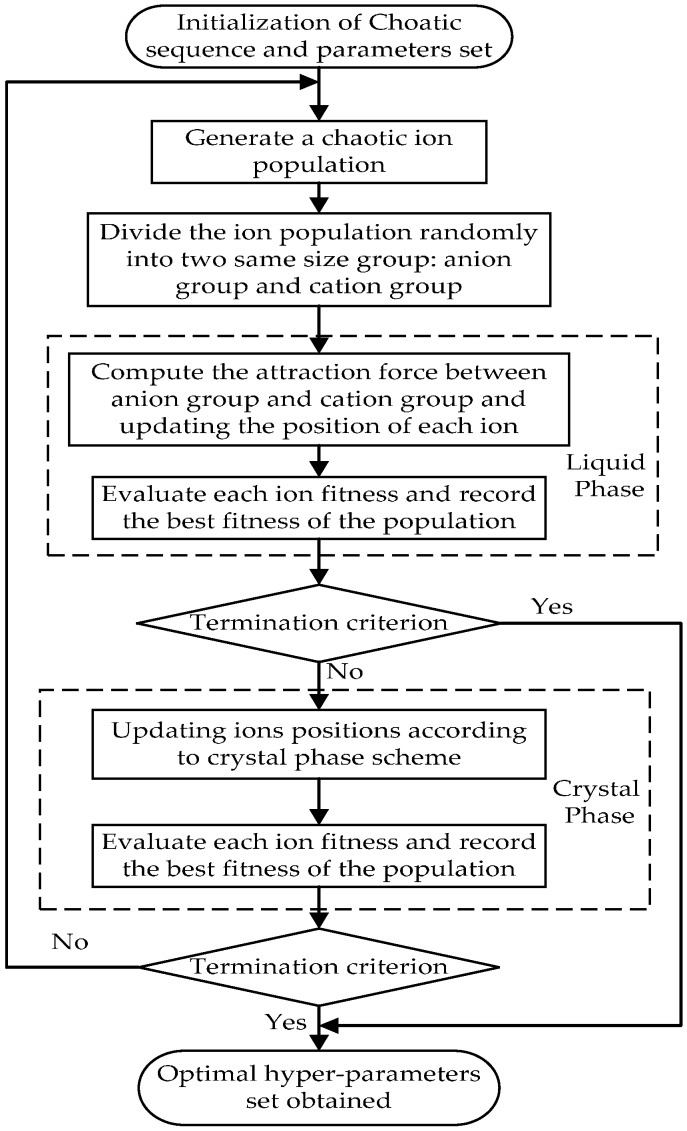
Chaotic ions algorithm optimized hybrid kernel least squares support vector machine (CIMA-LSSVM).

The flowchart of the chaotic ions motion algorithm optimized hybrid kernel least squares support vector machine (CIMA-LSSVM) is illustrated in Figure 3.

## 5. Data Calibration Experiment and Result Analysis

### 5.1. Data Calibration

**Figure 4:**
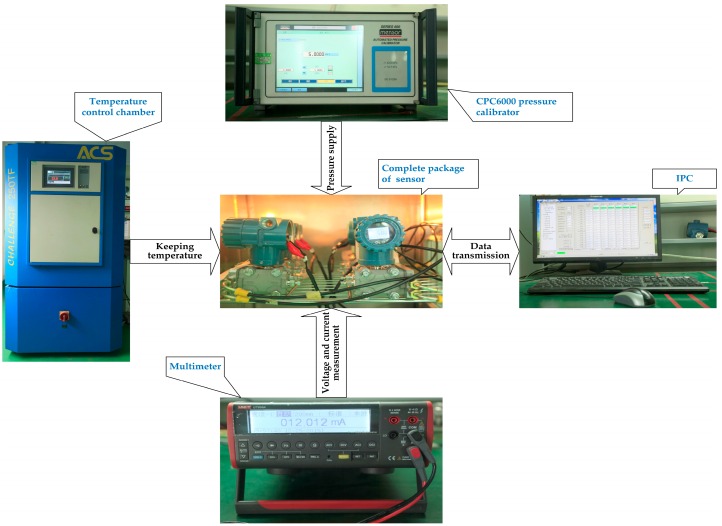
Set-up of calibration experiment.

A calibration experiment was performed on a grade of 0.065% pressure sensor without compensation [27] to provide data for modeling LSSVM. The measurement range of the pressure sensor used in this calibration is from −40 kPa to 40 kPa, and the working temperature range is from −20 °C to 70 °C. The measured temperature and the sensor output are converted by an analog-digital (A/D) converter, which were marked as T_AD_ and U_AD_. The standard input pressure denoted as *P* is specified with a step of 5 kPa from −40 kPa to 40 kPa and exerted by a CPC6000 pressure calibrator. Data calibration is made of eleven different temperature preservation processes at different temperature levels of −20 °C, −10 °C, 0 °C, 10 °C, 20 °C, 27 °C, 35 °C, 40 °C, 50 °C, 60 °C and 70 °C. Each temperature preservation process includes a temperature regulation process in temperature control chamber from present temperature to specified temperature and a thermal equilibrium process in the packaged sensor. Every temperature preservation process lasts about 3 h. One of six packaged sensors is chosen to accomplish the temperature compensation modeling. The experiment setup is demonstrated in Figure 4.

**Figure 5:**
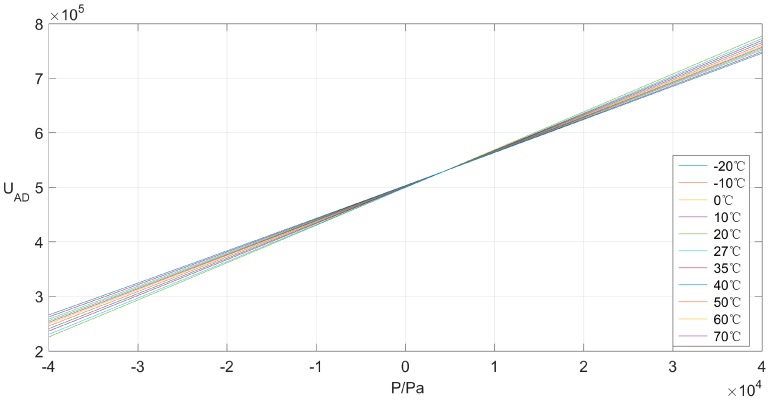
Pressure sensor’s output at different temperature.

**Figure 6:**
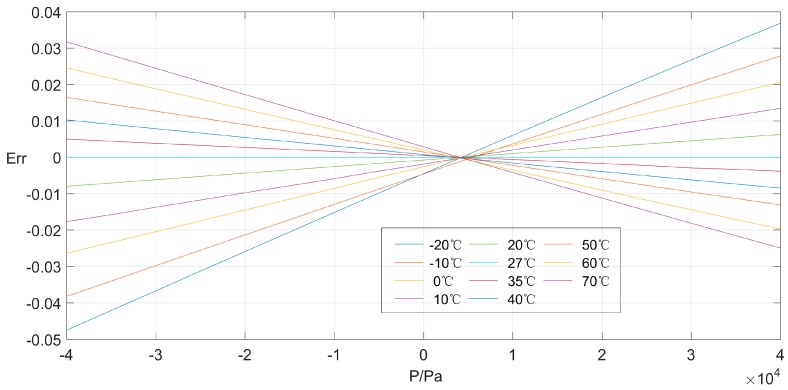
Relative error of pressure sensor with the benchmark data at 27 °C.

The calibration data obtained from the experiment is tabulated as Table 1. As shown in Figure 5, the sensor’s output performance without compensation varies notably, since it is influenced obviously by temperature variation. Furthermore, the maximum value of all relative errors (Err) are depicted in Figure 6, which reaches 4.75% with respect to the sensor’s output at the benchmark temperature of 27 °C. The analysis consequence of calibration data, therefore, forces sensor users to develop an efficient temperature compensation scheme, which could alleviate and even eliminate the dramatic temperature effect to satisfy the application requirement.

### 5.2. Data Preprocessing

Features in the calibration experiment have mismatched levels of magnitude, which would give rise to difficulties in modeling and generate an undesirable result. Thus, the experiment sample should be normalized into [−1, 1] according to the form as:

(24) $${\hat{x}}_{i}=\frac{x_{i}-\left(x_{imax}-x_{imin}\right)/2}{\left(x_{imax}-x_{imin}\right)/2},$$

where $x_{i}$ is the ith variable of a data point, and $x_{imax}$ and $x_{imin}$ are the upper limitation and lower limitation of *i*th variable, respectively. After normalizing the experiment data, a training set and a testing set should be partitioned before modeling. From the algorithm robustness viewpoint, a training set should be comprised of one-third of data points that are randomly selected from the sample, and the rest of the sample should construct the corresponding testing set. In the context of engineering, a training set may consist of all standard pressure values at several temperature levels and rest of the sample forming the testing set. These two sample partition strategies are both discussed in this article.

### 5.3. Modeling Temperature Compensation and Result Analysis

In order to validate the effectiveness of the proposed algorithm, several methods include SVM with RBF kernel [28], particle swarm optimization optimized RBF kernel SVM (PSO-RBF-SVM) [29], particle swarm optimization optimized RBF kernel LSSVM (PSO-RBF-LSSVM) [30], particle swarm optimization optimized hybrid kernel LSSVM (PSO-Hybrid-LSSVM), and ions motion algorithm optimized hybrid kernel LSSVM (IMA-Hybrid-LSSVM) are investigated. Because optimization techniques discussed in this article are all population-based algorithms, the hyper-parameters set (C,t,p,σ,λ) is defined as an individual in any population. The fitness of each algorithm is the mean squared error (MSE) of all fitting errors between the ideal pressure values and the compensated pressure values. The parameters of each algorithm are demonstrated in Table 2. The whole coding is implemented by developing the LS-SVMlab toolbox and libsvm toolbox on the MATLAB (2015b) platform (The Mathworks, Inc., Natick, MA, USA) [31,32]. Each algorithm is performed on 50 runs to avoid compensation results of chance and cross validation is adopted to prevent overfitting [33].

#### 5.3.1. Random Partition of the Sample

**Figure 7:**
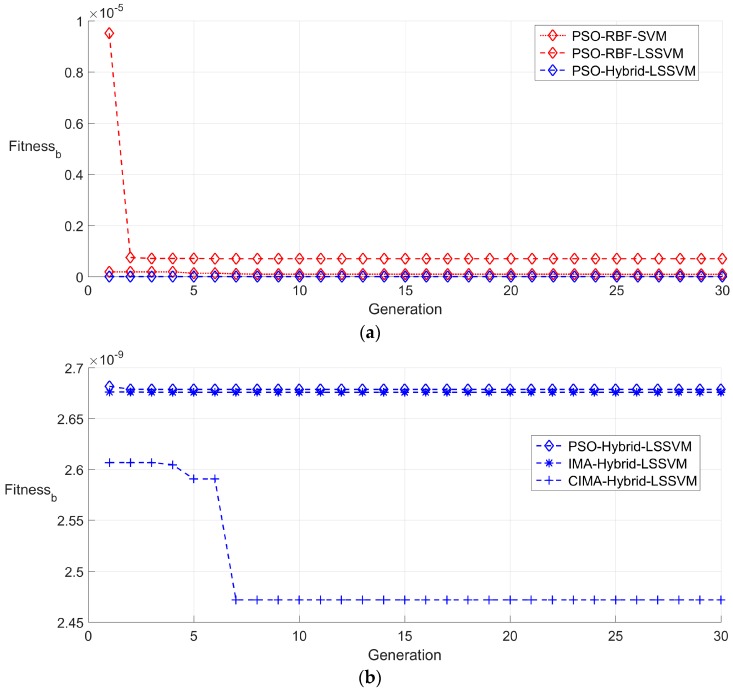
Best fitness of training set by random partition in 30 generations. (**a**) Best fitness obtained by particle swarm optimization optimized RBF kernel SVM (PSO-RBF-SVM), particle swarm optimization optimized RBF kernel LSSVM (PSO-RBF-LSSVM) and particle swarm optimization optimized hybrid kernel LSSVM (PSO-Hybrid-LSSVM); and (**b**) best fitness obtained by particle swarm optimization optimized hybrid kernel LSSVM (PSO-Hybrid-LSSVM), ions motion algorithm optimized hybrid kernel LSSVM (IMA-Hybrid-LSSVM) and chaotic ions motion algorithm optimized hybrid kernel LSSVM (CIMA-Hybrid-LSSVM).

A training set and a testing set are obtained on the basis of the random partition strategy. A typical searching process of the best population fitness (Fitness_b_) during the training period is demonstrated in Figure 7. Some conclusions can be drawn from the observation of Figure 7; firstly, the PSO-RBF-SVM algorithm converges faster than PSO-RBF-LSSVM, but the latter has a more desirable final best fitness than the former. This phenomenon indicates that, even with the same kernel function and identical optimization algorithm, the simplicity of LSSVM still provides more searching operations than SVM within the same time limit; secondly, it can be noted that the comprehensive advantage of hybrid kernel enables PSO-Hybrid-LSSVM to have a better final best fitness than PSO-RBF-LSSVM; thirdly, the performance of PSO-Hybrid-LSSVM and IMA-Hybrid-LSSVM is similar to each other whether on the convergence speed or the achieved final best fitness, this seems that any optimization algorithm devotes almost equally to the searching ability when employed the identical kernel function and fitting model. The randomness and the ergodic quality of the chaotic searching process guarantee the exploring and exploiting ability in the chaotic ions motion algorithm optimized hybrid kernel LSSVM (CIMA-Hybrid-LSSVM); thus, it can achieve the most satisfied searching performance; lastly, the best fitness at first iteration shows that the compensation model with hybrid kernel function offers a more elegant performance than those without it.

The validation standard of the compensation algorithm is the absolute relative error, which takes the form of:

(25) $$e_{ir}=\left|\frac{P_{i}^{\prime }-P_{i}}{P_{FS}}\right|\times 100\%,$$

where *i* is the standard pressure index, $P^{\prime }$ is the compensated pressure, *P* is the real standard pressure, and *P_FS_* is the full scale of the sensor.

Because the optimal hyper-parameters obtained on every run are different from each other, averaged values of training set error and testing set error are considered to balance the compensation results over all runs. Five performance indices as minimum (min), maximum (max), mean (mean), variance (var) of the averaged training set errors (e_ir_), and the mean training time during training period (MTT) are presented to evaluate the averaged performance of every approach, and the details are summarized in Table 3. As the compensation results, the first four indices of testing errors are summarized in Table 4. By analyzing the data in Table 3 and Table 4, it can be inferred that SVM and PSO-RBF-SVM can only provide a really limited improvement of the sensor performance; however, by utilizing algorithms within the framework of LSSVM, the compensation model can achieve a more satisfactory performance. Furthermore, optimization methods with hybrid kernel hold both good learning and generalization ability. The presented method takes full advantage of chaotic searching, hybrid kernel and LSSVM framework to achieve the most desirable compensation results at the lowest time cost.

#### 5.3.2. Fixed Partition of the Sample

**Figure 8:**
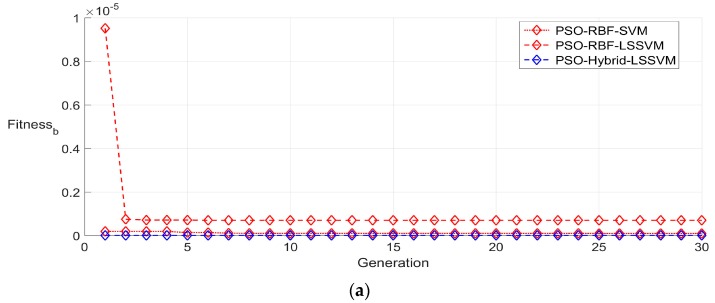

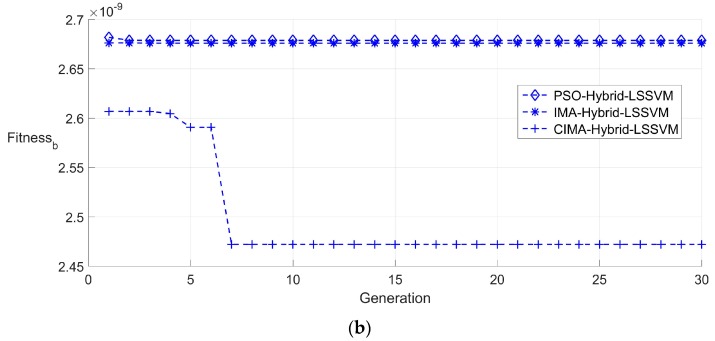
Best fitness of training set by fixed partition in 30 generations. (**a**) Best fitness obtained by particle swarm optimization optimized RBF kernel SVM (PSO-RBF-SVM), particle swarm optimization optimized RBF kernel LSSVM (PSO-RBF-LSSVM) and particle swarm optimization optimized hybrid kernel LSSVM (PSO-Hybrid-LSSVM); and (**b**) best fitness obtained by particle swarm optimization optimized hybrid kernel LSSVM (PSO-Hybrid-LSSVM), ions motion algorithm optimized hybrid kernel LSSVM (IMA-Hybrid-LSSVM) and chaotic ions motion algorithm optimized hybrid kernel LSSVM (CIMA-Hybrid-LSSVM).

In order to reduce the considerable engineering calibration time cost, data at four temperature levels including −20 °C, 10 °C, 40 °C and 70 °C are adopted as training sets in fixed partition strategy. The parameter settings of all algorithms are the same as the random partition strategy. A typical searching process of the best population fitness (Fitness_b_) of all compensation methods during the training period is demonstrated in Figure 8. It may be observed that the characteristics of compensation performance among all six algorithms are similar to the analysis mentioned in random partition strategy, but the convergence values of the best fitness are improved to a certain extent for the fixed partition strategy may provide more topology information about the training set.

The relative errors of the fixed partitioned training set and testing set are also averaged after 50 runs. The training results are summarized in Table 5. By comparing with the data in Table 3, it can be seen that all indices of compensation approaches in the framework of LSSVM with hybrid kernel do not change a lot, while other compensation approaches appear to have an obvious mutation, which proves the robustness of LSSVM. It can be also noted that the mean training time of PSO-RBF-SVM reduces from 210.7 s to 10.71 s for the training data topology formed by fixed partition is more suitable for PSO-RBF-SVM than by random partition.

The testing data compensation results are listed in Table 6. The reason for the difference between the compensation results of two partition strategies is that random partition intrinsically acquires more prior knowledge about the model by covering sample space more uniformly than fixed partition does. It also confirms that the formation of the training set is critical for the modeling in comparison with random partition strategy.

**Figure 9:**
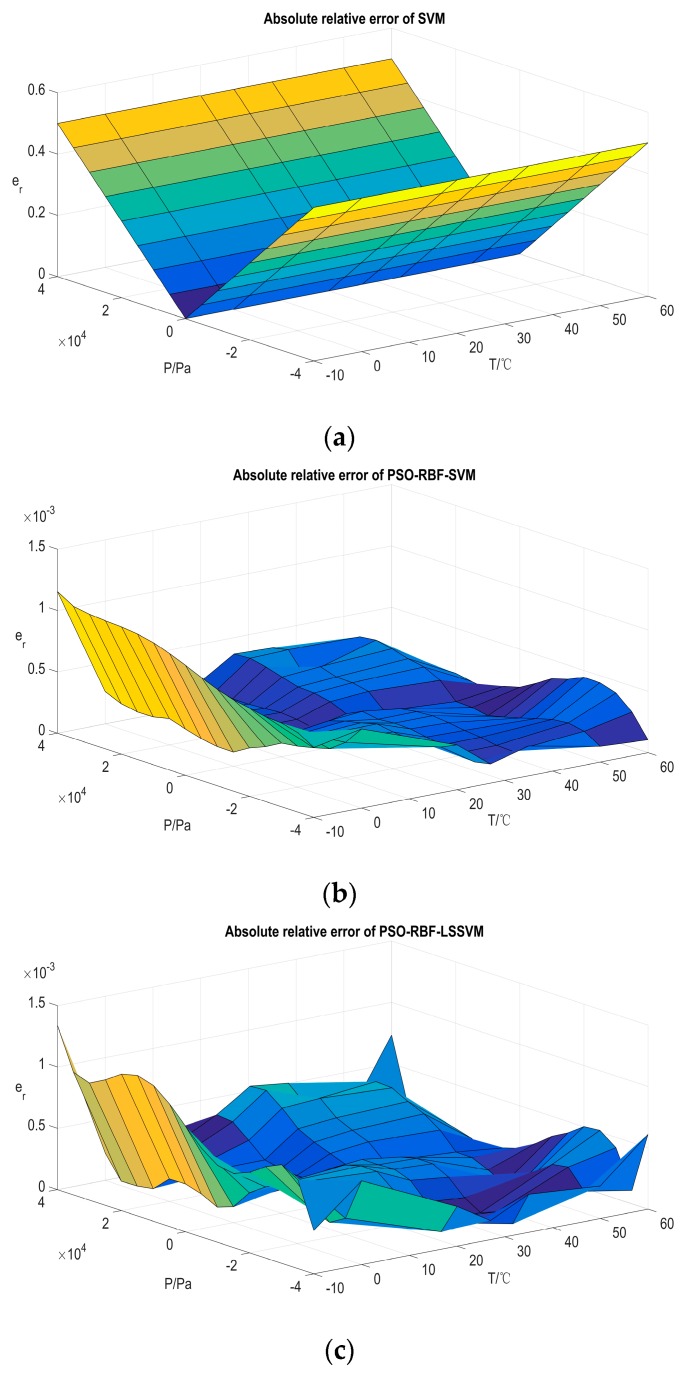

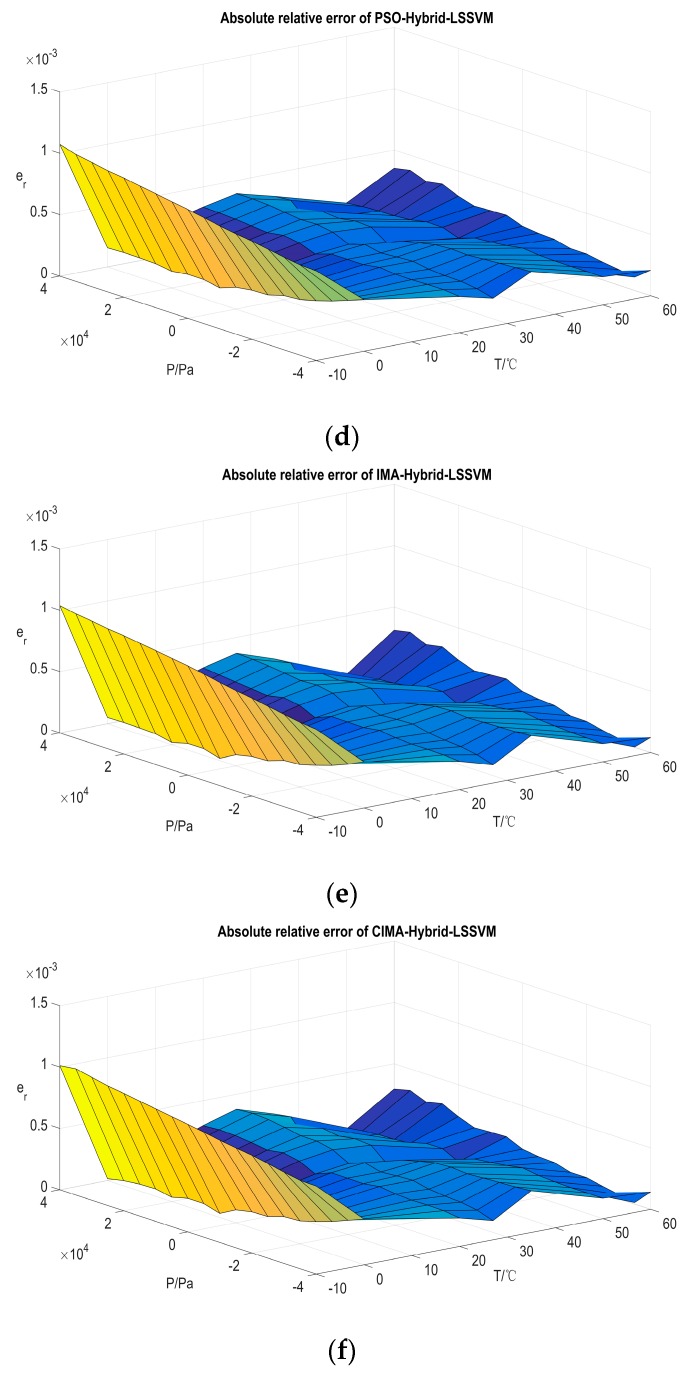
Compensation results on averaged testing set by fixed partition. (**a**) Compensation results obtained by SVM; (**b**) compensation results obtained by PSO-RBF-SVM; (**c**) compensation results obtained by PSO-RBF-LSSVM; (**d**) compensation results obtained by PSO-Hybrid-LSSVM; (**e**) compensation results obtained by IMA-Hybrid-LSSVM; and (**f**) compensation results obtained by CIMA-Hybrid-LSSVM.

The compensation results of all averaged testing data, marked as $e_{r}$, are illustrated in Figure 9. The temperature compensation errors of algorithms with hyper-parameter optimization show some randomness, which means that these compensation approaches fit the actual input–output model of the piezoresistive pressure sensor appropriately. The maximum compensation error occurs at −10 °C, as low temperature makes the characteristics of the packaged sensor more complex. Except for the maximum $e_{r}$ of the presented compensation method being slightly higher than that of the IMA-Hybrid-LSSVM, the absolute relative errors ($e_{r}$) on the rest points in the test set are the smallest. The modeling results of the experiment data with either random partition or fixed partition sufficiently prove the superiority of the proposed compensation method and also its satisfactory engineering capability.

## 6. Conclusions

A temperature compensation approach within the framework of LSSVM is presented in this research. Taking the full advantage of both local kernel and global kernel, a hybrid kernel function is constructed to balance the learning ability and generalization ability. The chaotic ions motion inspired algorithm is applied to search for the optimal hyper-parameters of LSSVM. Sample data for modeling LSSVM is acquired through a calibration experiment. The presented temperature compensation scheme avoids the restricted character of the single kernel function and refines the hyper-parameter searching procedure. The compensation results indicate that it is a highly effective compensation method both in the context of the algorithm robustness and engineering.

## Figures and Tables

**Table 1:** Data calibration.

P (Pa)	T (°C)										
	−20	−10	0	10	20	27	35	40	50	60	70
	T_AD_										
	46113	45715	45268	44875	44445	44066	43834	43559	43255	42833	42449
	U_AD_										
−40000	225163	229882	235926	240350	245293	249365	251904	254585	257738	261883	265502
−35000	258948	263075	268467	272361	276787	280403	282638	285002	287807	291422	294694
−30000	292891	296416	301166	304587	308434	311591	313534	315581	318035	321166	324036
−25000	326975	329900	334016	336931	340227	342921	344579	346307	348415	351061	353526
−20000	361190	363517	367004	369421	372163	374394	375763	377152	378937	381095	383158
−15000	395535	397260	400131	402004	404232	406003	407086	408178	409595	411274	412937
−10000	429974	431111	433352	434705	436398	437712	438518	439281	440352	441555	442817
−5000	464508	465059	466694	467572	468680	469525	470046	470503	471221	471962	472818
0	499132	499093	500112	500490	501053	501453	501666	501827	502188	502462	502916
5000	533794	533200	533579	533466	533475	533419	533347	533205	533214	533028	533077
10000	568569	567362	567163	566524	566019	565509	565185	564690	564373	563699	563370
15000	603351	601564	600769	599619	598591	597628	597024	596217	595555	594429	593697
20000	638130	635788	634383	632731	631170	629766	628882	627762	626757	625174	624052
25000	672932	670020	668039	665862	663787	661936	660781	659345	657996	655939	654454
30000	707712	704235	701674	699016	696402	694107	692600	690934	689248	686760	684868
35000	742451	738421	735281	732121	728999	726261	724565	722513	720489	717537	715282
40000	777129	772552	768840	765221	761560	758380	756415	754060	751701	748336	745668

**Table 2:** Parameters setting of algorithms.

Parameters	PSO [29]	PSO [30]	IMA
swarm size	50	50	50
maximum iteration number	30	30	30
fitness	MSE	MSE	MSE
maximum weight	0.9	0.9	
minimum weight	0.4	0.4	
social factor	[1, 3]	2	
cognitive factor	[1, 3]	2	

PSO-particle swarm optimization; IMA-ions motion algorithm.

**Table 3:** Compensation results of training set by random partition.

Temperature Compensation Methods	e_ir_ (min)	e_ir_ (max)	e_ir_ (mean)	e_ir_ (variance)	MTT (s)
SVM	4.814 × 10^−3^	5.026 × 10^−3^	4.973 × 10^−3^	2.237 × 10^−9^	1.499 × 10^−2^
PSO-RBF-SVM	5.550 × 10^−4^	1.334 × 10^−3^	9.673 × 10^−4^	3.385 × 10^−9^	2.107 × 10^2^
PSO-RBF-LSSVM	2.199 × 10^−4^	3.556 × 10^−4^	2.703 × 10^−4^	1.080 × 10^−9^	1.200 × 10^−2^
PSO-Hybrid-LSSVM	2.586 × 10^−4^	3.588 × 10^−4^	3.126 × 10^−4^	4.996 × 10^−10^	1.478 × 10^−2^
IMA-Hybrid-LSSVM	1.593 × 10^−4^	2.401 × 10^−4^	1.933 × 10^−4^	3.776 × 10^−10^	1.418 × 10^−2^
CIMA-Hybrid-LSSVM	1.353 × 10^−5^	4.413 × 10^−5^	2.497 × 10^−5^	5.258 × 10^−11^	1.392 × 10^−2^

**Table 4:** Compensation results of testing set by random partition.

Temperature Compensation Methods	e_ir_ (min)	e_ir_ (max)	e_ir_ (mean)	e_ir_ (variance)
SVM	2.106 × 10^−1^	3.197 × 10^−1^	2.648 × 10^−1^	5.562 × 10^−4^
PSO-RBF-SVM	5.924 × 10^−4^	2.187 × 10^−3^	1.235 × 10^−3^	1.085 × 10^−7^
PSO-RBF-LSSVM	2.422 × 10^−4^	6.706 × 10^−4^	4.011 × 10^−4^	6.286 × 10^−9^
PSO-Hybrid-LSSVM	2.733 × 10^−4^	5.165 × 10^−4^	3.687 × 10^−4^	1.494 × 10^−9^
IMA-Hybrid-LSSVM	1.841 × 10^−4^	3.424 × 10^−4^	2.517 × 10^−4^	9.659 × 10^−10^
CIMA-Hybrid-LSSVM	1.155 × 10^−4^	2.559 × 10^−4^	1.770 × 10^−4^	1.104 × 10^−9^

**Table 5:** Compensation results of training set by fixed partition.

Temperature Compensation Methods	e_ir_ (min)	e_ir_ (max)	e_ir_ (mean)	e_ir_ (variance)	MTT (s)
SVM	3.272 × 10^−4^	5.220 × 10^−3^	4.722 × 10^−3^	1.227 × 10^−6^	1.507 × 10^−2^
PSO-RBF-SVM	4.343 × 10^−5^	3.248 × 10^−4^	1.850 × 10^−4^	5.467 × 10^−9^	10.71 × 10^1^
PSO-RBF-LSSVM	8.946 × 10^−6^	1.111 × 10^−3^	1.927 × 10^−4^	3.692 × 10^−8^	2.448 × 10^−2^
PSO-Hybrid-LSSVM	1.085 × 10^−4^	1.564 × 10^−4^	1.251 × 10^−4^	1.159 × 10^−10^	2.549 × 10^−2^
IMA-Hybrid-LSSVM	4.095 × 10^−7^	6.063 × 10^−5^	1.444 × 10^−5^	1.844 × 10^−10^	2.641 × 10^−2^
CIMA-Hybrid-LSSVM	9.921 × 10^−6^	6.401 × 10^−5^	2.029 × 10^−5^	1.210 × 10^−10^	2.568 × 10^−2^

**Table 6:** Compensation results of testing set by fixed partition.

Temperature Compensation Methods	e_ir_ (min)	e_ir_ (max)	e_ir_ (mean)	e_ir_ (variance)
SVM	3.414 × 10^−4^	5.003 × 10^−1^	2.647 × 10^−1^	2.387 × 10^−2^
PSO-RBF-SVM	7.647 × 10^−5^	1.154 × 10^−3^	3.290 × 10^−4^	6.368 × 10^−8^
PSO-RBF-LSSVM	1.953 × 10^−5^	1.338 × 10^−3^	3.549 × 10^−4^	7.548 × 10^−8^
PSO-Hybrid-LSSVM	1.082 × 10^−4^	1.074 × 10^−3^	3.555 × 10^−4^	5.374 × 10^−8^
IMA-Hybrid-LSSVM	1.831 × 10^−6^	1.038 × 10^−3^	3.007 × 10^−4^	6.565 × 10^−8^
CIMA-Hybrid-LSSVM	1.224 × 10^−5^	1.066 × 10^−3^	3.056 × 10^−4^	6.427 × 10^−8^
